# Mixed Infections and Hybridisation in Monogenean Parasites

**DOI:** 10.1371/journal.pone.0039506

**Published:** 2012-07-11

**Authors:** Bettina Schelkle, Patricia J. Faria, Mireille B. Johnson, Cock van Oosterhout, Joanne Cable

**Affiliations:** 1 School of Biosciences, Cardiff University, Cardiff, United Kingdom; 2 School of Environmental Sciences, University of East Anglia, Norwich Research Park, Norwich, United Kingdom; Institut Pasteur, France

## Abstract

Theory predicts that sexual reproduction promotes disease invasion by increasing the evolutionary potential of the parasite, whereas asexual reproduction tends to enhance establishment success and population growth rate. Gyrodactylid monogeneans are ubiquitous ectoparasites of teleost fish, and the evolutionary success of the specious *Gyrodactylus* genus is thought to be partly due to their use of various modes of reproduction. *Gyrodactylus turnbulli* is a natural parasite of the guppy (*Poecilia reticulata*), a small, tropical fish used as a model for behavioural, ecological and evolutionary studies. Using experimental infections and a recently developed microsatellite marker, we conclusively show that monogenean parasites reproduce sexually. Conservatively, we estimate that sexual recombination occurs and that between 3.7–10.9% of the parasites in our experimental crosses are hybrid genotypes with ancestors from different laboratory strains of *G. turnbulli*. We also provide evidence of hybrid vigour and/or inter-strain competition, which appeared to lead to a higher maximum parasite load in mixed infections. Finally, we demonstrate inbreeding avoidance for the first time in platyhelminths which may influence the distribution of parasites within a host and their subsequent exposure to the host's localized immune response. Combined reproductive modes and inbreeding avoidance may explain the extreme evolutionary diversification success of parasites such as *Gyrodactylus*, where host-parasite coevolution is punctuated by relatively frequent host switching.

## Introduction

The widespread occurrence of sex is usually attributed to the fact that recombination generates new gene combinations, thereby increasing the rate of adaptive evolution whilst negating Muller’s ratchet and the associated irreversible accumulation of deleterious mutations [1]. However, if sexual reproduction prevails in populations with small effective population sizes, consanguineous mating over several generations will result in severe inbreeding, potentially leading to inbreeding depression [2]. The deleterious effects of inbreeding are predicted to be least noticeable in populations with a long history of inbreeding, as this tends to purge deleterious mutations in the population [3]–[6]. Conversely, the beneficial effects of outcrossing are expected to be most pronounced in the most inbred populations [2]. This prediction is based on the assumption that in highly-inbred populations at least some loci will have been fixed for recessive deleterious alleles by drift, and that outcrossing will restore these loci to a heterozygous state [7]. Hybridization may result in heterosis (or hybrid vigour), an increase in fitness in the F1 offspring [2]. However, the benefits of outcrossing and hybrid vigour tend to decline in subsequent generations due to the breakdown of co-adapted gene complexes and epistatic gene interactions [2], [8].

Among micro- and macroparasites, hybridization has been observed at an intraspecific [9]–[11] as well as interspecific level [12]–[14]. It can lead to the emergence of new diseases [15], [16], with hybrid origins and/or current interbreeding events shown for viruses (e.g. Spanish flu, human rotavirus) [17], [18], bacteria (e.g. *Haemophilus influenza*) [19], fungi and oomycetes [20], protozoa (e.g. *Leishmania infantum*, *Trypanosoma cruzi*) [11], [21] and various schistosome species [22], [23]. Immediate consequences of hybridization may involve increased pathogen fecundity, infectivity, virulence and transmission rates, wider host spectra, a shorter maturation time and phenotypic changes [15], [16]. For instance, hybrid vigour in F1 offspring of *Leishmania infantum*/*L. major* crosses led to increased resistance to immunity in an atypical vector [24]. However, the fitness advantages of hybridization are typically short-lived; most laboratory hybrids that show hybrid vigour lose their fitness in subsequent generations [8], [25], [26].

For gyrodactylids (a specious group of monogeneans), there is phylogenetic evidence that co-infecting species may hybridize before and after host switches [14], [27]–[29]. These ectoparasitic monogeneans are ubiquitous on teleost fish [30] and are largely transmitted between fish by direct host-host contact with a single parasite being sufficient to seed a population [31]. Most give birth to live young, which are already pregnant when born, a phenomenon termed hyperviviparity [32]. The first born daughter is derived asexually by mitotic division when the mother is still an embryo; the second daughter can be produced by automictic parthenogenesis, and all other daughters (up to five in total recorded to date) are either produced via parthenogenesis or potentially sexual reproduction [31]. Hyperviviparity in combination with extreme progenesis allows these parasites to produce offspring in as little as 24 h (e.g. *G. turnbulli*) [33] resulting in explosive population growth. It has been hypothesized that during epidemic population growth, sexual reproduction is more common than parthenogenesis due to crowding effects [34], [35]. However for all monogeneans, the occurrence of sexual recombination has been assumed (e.g. [28]), but never actually proven.

In this study, we used a microsatellite marker to confirm sexual reproduction in monogeneans. This methodology was then applied to assess the effects of hybridizing three *G. turnbulli* strains that have been isolated and inbred in the laboratory for 1, 8 and 12 years (circa 2×10^2^ to 2×10^3^ generations; assuming 2 days/generation). We then (conservatively) estimate the proportion of parasites that have a hybrid origin. By comparing the infection trajectories, maximum parasite burdens and duration of infections in parental and mixed populations, we provide evidence for hybrid vigour and/or inter-strain competition. By analysing time-to-infection for parasites transmitting to fish infected with same-strain or different-strain individuals, we show inbreeding avoidance behaviour within strains.

## Materials and Methods

This work was conducted using the guppy (*Poecilia reticulata*) – *Gyrodactylus turnbulli* model system with all procedures conducted under UK Home Office licence (PPL 30/2357) regulations and approved by the Cardiff University Ethics Committee.

### Source of Animals and Infection Procedures

Guppies (*Poecilia reticulata*, Peters) were originally isolated from a wild population from the River Tunapuna (20P, 678513E, 1177415N), Trinidad, and thereafter maintained in laboratory cultures for ca. 8 years. The fish were maintained at 25±1°C with a 12 h light : 12 h dark cycle at a density of approximately 120 fish per 40L aquarium, and fed at least twice daily on Aquarian® fish flakes plus *Artemia* and/or *Daphnia* spp. in addition to bloodworm once a week.

Three different *Gyrodactylus turnbulli* strains were used: *Gt3* (isolated from a pet shop guppy in Nottingham in 1997), *Gt1* (isolated from wild guppies in the Lower Aripo River, Trinidad, in November 2001) and *Gt8* (isolated from a pet shop guppy in Cardiff in March 2008), which are routinely maintained at Cardiff University at 25±0.5°C on ornamental guppies. In this study, crosses are made between these lines and we use the term ‘hybrid’ or ‘hybrid genotype’ *sensu stricto*: “(1) a progeny of a cross between parents of different genotype; (2) heterozygote” [36].

Experimental infections utilized donor guppies carrying >20 parasites per fish from either of the three laboratory strains (*Gt1, Gt3* and *Gt8*) and which were anaesthetized with 0.02% Tricaine Methanesulfonate (MS222, PharmaQ, UK). Guppies that were naïve to these parasites were also anaesthetized, sexed, measured and infected by bringing the infected donor guppy into contact with the recipient until the gyrodactylid moved from one fish to the other. To ensure infection by one parasite strain was not biased by prior exposure to another strain, half of the fish were first infected with one strain (i.e. first infection), then the other (i.e. second infection) so avoiding any ‘priority effects’. Extreme care was taken to prevent cross-infection of individual fish with the wrong strain of parasite by changing anaesthetic and glassware after each infection procedure. At various stages during Experiments 1 and 2, parasites were removed from anesthetized hosts with fine watchmaker’s forceps and stored in 100% ethanol for subsequent molecular analysis.

### Experiment 1: Sexual Recombination in Gyrodactylids

To maximise our chances of detecting hybrid genotypes and to achieve high parasite infrapopulations (assuming sex may only occur at high parasite densities) [34] we began the infection experiment with 10 parasites on each fish. In total, 24 guppies were infected with ten monogeneans each, five from two different parasite strains, resulting in 12 fish infected with *Gt3* and *Gt1* (parasite line *Gt3*×*Gt1*) and 12 fish infected with *Gt8* and *Gt1* (parasite line *Gt8*×*Gt1*). Fish were maintained in pairs in 1L plastic pots to which two naïve fish were added the subsequent day (D1). Thereafter, water was changed every day and any deceased guppies were replaced with naïve fish. On D14, the parasite cultures were screened and the most heavily infected fish (1–2 fish per culture, infected with over 100 parasites) were euthanized with an overdose of MS222 and fixed in ethanol. A sample of parasites from these highly infected, fixed fish were removed with entomological pins and stored individually in fresh 100% ethanol.

### Experiment 2: Hybrid Fitness and/or Inter-strain Competition

In this experiment, recipient fish (n = 101) were infected with either two parasites of the same strain (paternal parasite lines: *Gt1*×*Gt1*, *Gt3*×*Gt3* and *Gt8*×*Gt8*; hereafter the “pure parasite population”) or two parasites from different strains (hybrid parasite lines: *Gt3*×*Gt1* and *Gt8*×*Gt1*, hereafter the “mixed parasite population”). The time-to-infection success (i.e. the time a parasite took to transfer from the donor fish to the recipient fish during infections) [37] was recorded for both parasites. The infected recipient fish (*Gt3*: n = 10; *Gt1*: n = 11; *Gt8*: n = 11; *Gt3*×*Gt1*: n = 38; *Gt8*×*Gt1*: n = 31) were maintained individually in 1L pots.

On day 1 (D1) all fish were screened following procedures by Schelkle et al. [38] to check whether the infection had established (i.e. ≥2 parasites on a fish were regarded as successful establishment). From D1 onwards, all fish were screened every other day to follow the population dynamics of the parasites until the fish were screened clear for at least three times [38]. Additionally, on D7 half of the parasites (median: 6, range: 1–26) were removed with watchmaker’s fine forceps from the anaesthetized host and any monogeneans from fish containing mixed parasite population were fixed for subsequent molecular analysis to assess the presence of hybrid genotypes on individual fish. Halving the parasite load at this stage ensured we had samples to genotype while still allowing the fitness experiment to continue on a like-for-like basis (i.e. all parasite infrapopulations were halved).

### Microsatellite Genotyping

The three parasite strains *Gt1*, *Gt3* and *Gt8* were previously genotyped at four microsatellite loci [39]. However, only a single locus (TurB02) unambiguously discriminates between two of the three lines (i.e. was fixed for different alleles at two of the three lines). Hence, we use this locus to estimate the rate of sexual reproduction. *Gt3* and *Gt8* have different alleles to *Gt1* at the TurB02 locus, which allows us to detect potential hybrid genotypes between *Gt3* and *Gt1*, and *Gt8* and *Gt1*, but not *Gt3* and *Gt8*. Therefore, a mixed population of both latter strains was not included in this study.

In Experiment 1, a random sample of 48 gyrodactylids from 10 different mixed parasite cultures were screened to examine whether any hybrid (i.e. heterozygous) individuals were present, which would unambiguously establish sexual reproduction. In Experiment 2, 240 parasites (139 gyrodactylids isolated from 23 *Gt3*×*Gt1* infected fish, and 101 from 23 *Gt8*×*Gt1* infected fish) were successfully genotyped. Extraction was performed using the following lysis protocol: 50 µl of lysis buffer containing 1xTE, 0.45% Tween 20 and 9 µg of Proteinase K was added to a whole gyrodactylid and incubated at 65°C for 30 min and then denatured at 95°C for 10 min. PCR amplification was performed as described in [39] using 5 µl Master Mix (Qiagen multiplex PCR kit), 2 pmol of each microsatellite primer (TurB02: forward ACGAGTGACAATAAAGCTGG, reverse ATCAATAGTTGAATGG) and 2 µl of DNA following manufacturer’s instructions. The PCR cycling profile consisted of 15 min at 95°C, followed by 40 cycles of 40 s at 95°C, 90s at 52°C and 90 s at 72°C and a final extension for 30 min at 72°C. PCR products were loaded on an ABI3130XL using size standard ROX-350 (Applied Biosystems) and chromatograms were analyzed using PeakScanner version 1.0 (Applied Biosystems).

Parasites were identified as being of hybrid or parental strains based on their genotypes. All parental strains were homozygous for the locus used: *Gt3* and *Gt8* parental genotypes presented only allele 244 bp, whereas *Gt1* presented the allele 246 bp. This enabled us to distinguish hybrid genotypes in crosses with *Gt8*×*Gt1* and *Gt3*×*Gt1*. Since just one microsatellite locus was used to differentiate between heterozygous or homozygous individuals, non-detection of hybrid genotype monogeneans could have occurred for five reasons: (1) One of the parasite strains did not establish after infection, but the parasite infection was still recorded as being established. (2) No sexual reproduction occurred. (3) No sexually produced parasites were sampled. (4) Sexually reproduced parasites were homozygous for the microsatellite loci due to Mendelian segregation of alleles over several generations. (5) Sampled parasites were backcrossed individuals. Hence, hybridization rates quoted for both experiments below are significant underestimates and should be interpreted as a conservative estimate of sexual reproduction in our study system.

These five factors that potentially inflate the false-negative rate (i.e. the conclusion that no hybridisation occurred) also have implications for Experiment 2. Due to this potential non-detection of hybrid genotypes, analysis of the infection trajectories in Experiment 2 was only performed on replicates for which molecular analysis had revealed the presence of hybrid genotype parasites. This data was directly compared with the control treatments (parental strains only). Fish on which only parental genotypes were found, despite infection with two different strains, were discarded from the analysis. The rationale was that homozygous F2, F3, etc. and back-crossed individuals, and parasites that did not engage in sex, were indistinguishable from asexually produced individuals or non-establishment of one of the strains.

### Statistical Analysis

For Experiment 1, the occurrence of sex was assessed by scoring the presence of homozygous and heterozygous parasites on infected fish. Differences in cross-breeding success between parasite populations were assessed using a χ^2^ contingency table test in Minitab v15. A direct comparison of hybrid genotype frequency with Experiment 2 could not be conducted due to the differences in timing of gyrodactylid collection in the experiments.

We investigated whether there was evidence for hybrid vigour and/or inter-strain competition in mixed parasite population by comparing the infection intensity trajectories, maximum parasite burdens and duration of infections in parental and mixed populations in Experiment 2. We used a combination of general linear models (GLMs) and a general linear mixed model (GLMM) based on restricted maximum likelihood (REML) analyses in R and ASReml-R (R v2.9.2) [40]. All models used a Gaussian error distribution and an identity link function. For mixed parasite populations only data from fish with known hybrids (confirmed by molecular analysis; n = 12) were considered. These data were compared and contrasted to the data of the pure lines (n = 32). For infection intensity trajectories and maximum parasite burden data were normalised by a natural logarithmic, ln(x), transformation within the model; data for the day of peak parasite burden and parasite loss did not need transformation. For the GLMM, animal ID (to account for repeated parasite counts on the same fish) and days post-infection (with a spline to account for non-linear effects) were added to the random model. Standardized residual distributions were assessed visually with histograms and normality plots plus Shapiro-Wilk normality tests for all models. Initial model terms are presented in Table 1. All model terms were assessed as significant with α = 0.05 as critical value, and models were reduced with stepwise deletions (using the Log-Likelihood method for random terms in the GLMM). Finally, the minimal model for the GLMM was used to predict the infection trajectory on individual, isolated hosts.

**Table 1:** Model terms and interactions in the GLM and GLMM analyses used for Experiment 2.

Infection trajectory measure (model)	Dependent variable	Independent continuous (cont.)/categorical variable (cat.)
Infection intensity	Parasite intensity:	Parasite population (cat.)
trajectory (GLMM)	Ln (x) transformed	Fish length (cont.)
		Fish sex (cat.)
		Days post-infection (cont.)
		Animal ID (cat.)*
		Spline (days post-infection) (cont.)*
Maximum parasite	Maximum parasite burden:	Parasite population (cat.)
burden (GLM)	Ln (x) transformed	Fish length (cont.)
		Fish sex (cat.)
		Fish length X sex interaction
Day of maximum	Day of maximum parasite	Parasite population (cat.)
parasite burden (GLM)	burden	Fish length (cont.)
		Fish sex (cat.)
		Fish length X sex interaction
Parasite loss (GLM)	Day of parasite loss	Parasite population (cat.)
		Fish length (cont.)
		Fish sex (cat.)
		Fish length X sex interaction

* random terms in GLMM.

We also examined inbreeding avoidance behaviour within strains by evaluating time-to-infection for parasites transmitting to fish infected with same-strain or different-strain individuals. Parasite time-to-infection was analysed with a survival analysis in R v2.9.2 using Cox’s Proportional Hazard and time-to-infection data for the second parasite to infect a fish. The initial model included two parameters: whether the second parasite infecting the recipient fish was a same or different strain individual compared to the first monogenean, as well as the strain origin of the second parasite. Parasite establishment was assessed with a binary logistic regression in Minitab using fish length, sex and parasite population as independent variables.

A χ^2^ contingency table test in Minitab or Fisher’s Exact Tests (available at http://www.physics.csbsju.edu/stats/contingency.html) [41] were used to detect (1) differences in recovery from parasite infection (i.e. screened clear from parasites on three subsequent screens) [38]; (2) percentage of fish carrying hybrid genotypes or not; and (3) percentage of hybrid genotypes in the two mixed populations on fish with hybrids.

Proportion of hybrid genotype gyrodactylids among all genotyped parasites and fish mortality were analysed using a binary logistic regression in Minitab including intended parasite population (pure or mixed) in the former and intended parasite population, fish length and sex in the latter.

## Results

### Sexual Reproduction and Hybridization

**Figure 1:**
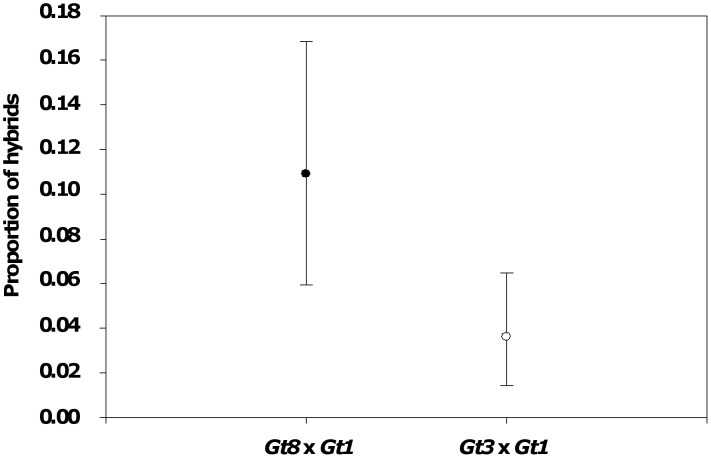
Frequencey of sexual reproduction in gyrodactylids. Proportion of hybrid parasites (±95% CI) for the *Gt3*×*Gt1* and *Gt8*×*Gt1* parasite populations in Experiment 2. A significantly higher proportion of hybrids were detected in the *Gt8*×*Gt1* than in the *Gt3*×*Gt1* cross.

Gyrodactylid strains interbred freely, with 37.5% and 34.6% hybrid genotypes recovered at D14 from the *Gt3*×*Gt1* and *Gt8*×*Gt1* parasite lines, respectively, in Experiment 1 (high density starting infection). There was no apparent difference in hybrid genotype frequency between crosses (Contingency Table Test: χ^2^ = 0.201, d.f. = 1, P = 0.654). In Experiment 2 (low density starting infection), twelve fish were confirmed to carry hybrid genotype parasites: five (out of 32) initially infected with *Gt3*×*Gt1* (15.6%) and seven fish (out of 31) initially infected with *Gt8*×*Gt1* (22.5%). In this experiment significantly more hybrid genotype parasites were detected for the *Gt8*×*Gt1* population (10.9%) than in the *Gt3*×*Gt1* population (3.7%; Binary Logistic Regression: G = 3.841, d.f. = 1, P = 0.05; Fig. 1).

### Fitness Effects of Mixed Strain Infections

**Figure 2:**
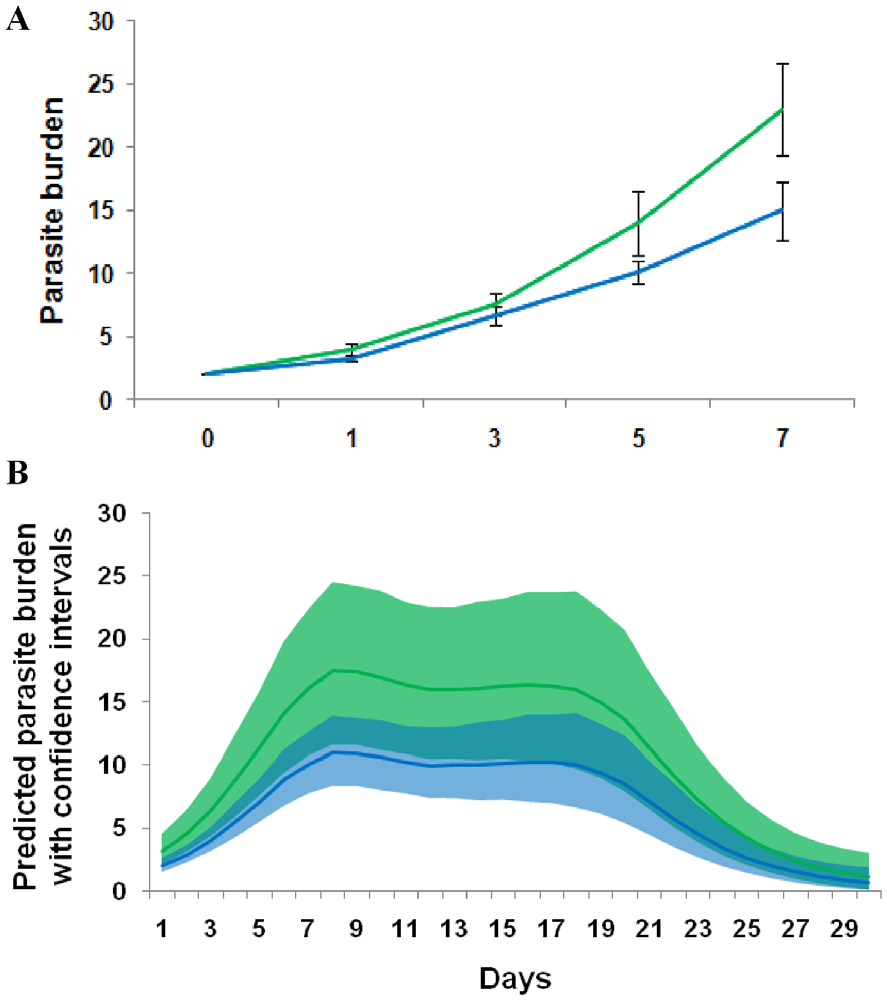
Infection trajectory of pure and mixed *Gyrodactylus turnbulli* populations on individual guppies (*Poecilia reticulata*). (A) Mean parasite intensity in pure (blue) and mixed parasite populations (green) (± Standard error of the mean; Experiment 2) in the initial phases of infection showing increased fitness in the mixed parasite population. Increased variation after day 7 is due to the onset of the host’s immune response and is not displayed in this graph. (B) Predicted mean parasite burden over time (controlled for fish standard length, ±95% Confidence Intervals) showing that increased parasite fitness in mixed parasite populations leads to faster population growth and higher maximum parasite burdens.

The GLM and GLMM minimal models for each dependent variable analysed in Experiment 2 are presented in Table 2. Mixed gyrodactylid populations exhibited hybrid vigour and/or inter-strain competition at a population level (GLMM: Wald = 5.805, d.f. = 1, p = 0.021). Fig. 2A shows the initial growth phase after which there is no clear pattern between parasite populations due to the onset of fish immunity. Hence, in Fig. 2B we use predicted data from the GLMM to visualise different infection trajectories.

**Table 2:** Minimal models for the GLM and GLMM analyses in Experiment 2.

**a. Infection trajectory. Model type: GLMM (random terms = fish ID, days post-infection)**			
Model term	Wald statistic (F)	d.f.	P
Parasite population	5.805	1	0.021
Fish length	12.7	1	0.001
Days post-infection	8.669	1	0.004
**b. Maximum parasite burden. Model type: GLM**			
Model term	Wald statistic (F)	d.f.	P
Parasite population	7.024	1, 40	0.012
Fish length	12.639	1, 40	>0.001
**c. Day of peak parasite burden. Model type: GLM**			
Model term	F statistics	d.f.	P
Parasite population	4.963	1, 32	0.033
Fish length	16.327	1, 32	>0.001
**d. Day of parasite loss. Model type: GLM**			
Model term	F statistics	d.f.	P
Fish length	13.343	1, 33	>0.001

For GLMs the term, then the residual degree of freedom are given.

**Figure 3:**
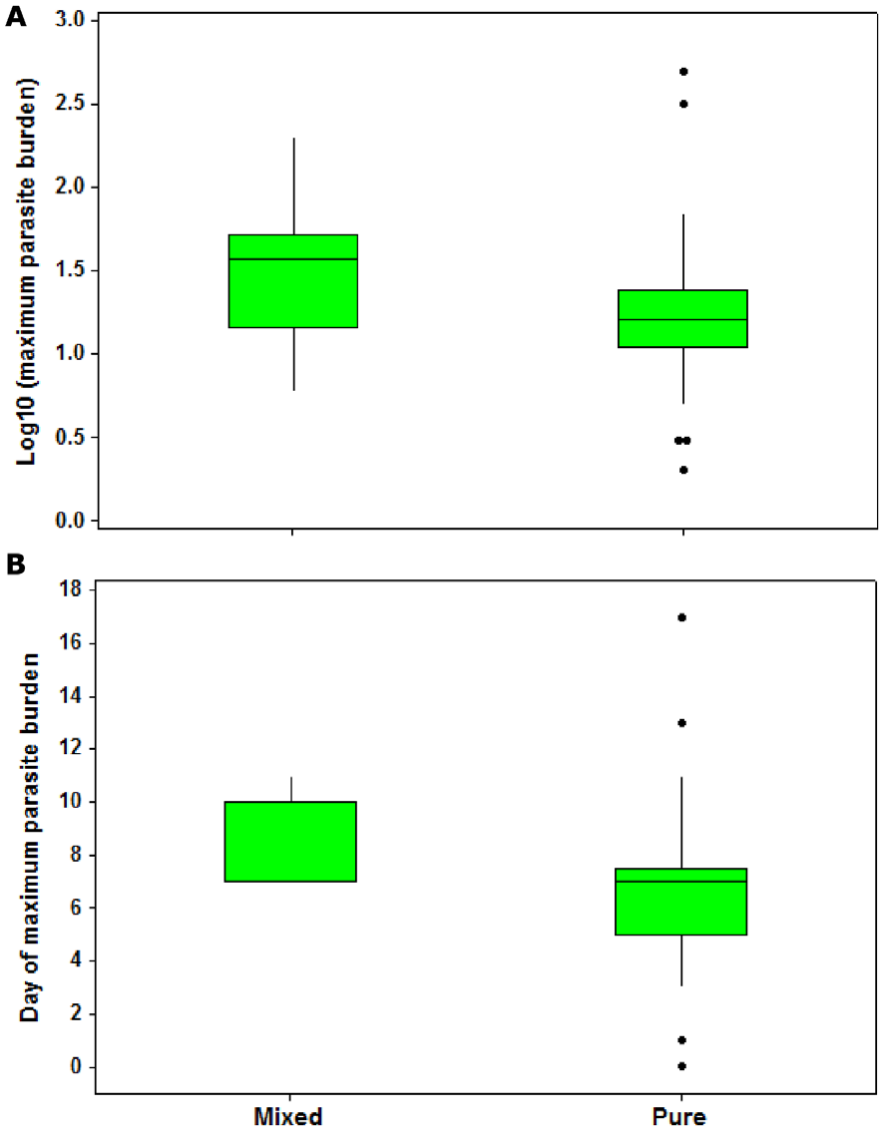
Maximum parasite burden and day of maximum parasite burden in pure and mixed parasite populations. (A) Median maximum parasite burden (log_10_ transformed) and (B) median day of maximum parasite burden. Outliers are represented by dots; the bars are the lower and upper limits; and the box represents the 1^st^ and 3^rd^ quartile with the median. Significant differences between mixed and pure parasite populations for both A (P = 0.012) and B (P = 0.033).

The apparent superiority of mixed infections was reflected in a higher parasite burden over time and increased maximum parasite burden, but not a longer duration of infection. Mixed infrapopulations reached their maximum parasite burden of 48.6±15.2 parasites per fish, which was significantly higher than the 44.2±18.3 parasites in pure parental infrapopulations (GLM: F_1,40_ = 7.024, p = 0.012; Fig. 3a). However, this peak of infection occurred later in mixed populations at 8.3±0.5 days compared to 6.7±0.6 days in pure populations (GLM: F_1,32_ = 4.963, p = 0.033; Fig. 3b). Parasite infrapopulations were lost at 14.6±1.2 and 15.5±1.5 days post-infection in mixed and pure populations, respectively (GLM: F_1,34_ = 1.103, p = 0.302).

The infection trajectory itself was also influenced by days post-infection (GLMM: Wald = 8.669, d.f. = 1, p = 0.004) and host length (GLMM: F_1_ = 12.7, p = 0.001). Larger hosts showed a higher maximum parasite burden (GLM: F_1,40_ = 12.639, p<0.001) which was reached slightly later (GLM: F_1,32_ = 16.327, p<0.001). Similarly, parasites were lost slightly later in large guppies (GLM: F_1,33_ = 13.343, p<0.001). There was no effect of host sex on maximum parasite burden, day of peak parasite burden, day of parasite loss or the infection trajectory.

### Parasite Infection, Establishment Success and Host Survival

**Figure 4:**
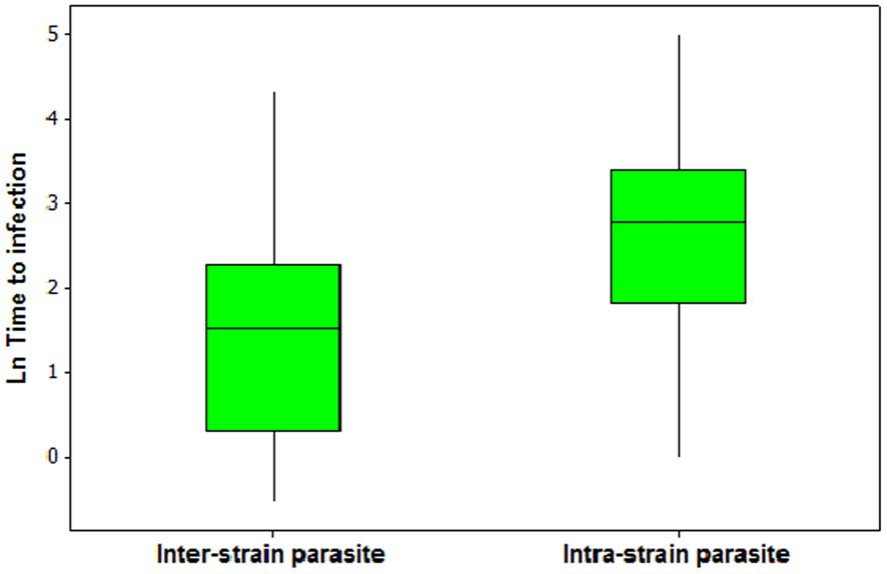
Time-to-infection for inter- and intrastrain infections. Time-to-infection (s; natural log transformed) was significantly lower for secondary parasites infecting a fish already infected with a different (inter-)strain parasite rather than a same (intra-)strain parasite individual. The bars are the lower and upper limits; and the box represents the 1^st^ and 3^rd^ quartile with the median.

In Experiment 2, infection success for parasites used to infect each fish was 100% for all replicates in all treatments. Time-to-infection of the second monogenean was significantly slower if the parasite on the fish (from the first infection) was from the same strain (25.3±2.2 s) than from a different strain (11.8±5.6 s; Survival analysis: z = −2.809, P = 0.005, n = 100; Fig. 4). Establishment success, measured on D1, increased with fish size (Binary Logistic Regression: G = 10.57, d.f. = 3, p = 0.014 for overall test; Z = −2.07, p = 0.039 for fish length, odds ratio 0.62 (95% CI: 0.39–0.98)), but was not affected by parasite population (pure: 96.9%, mixed: 89.9%; Z = 0.92, p = 0.359 for parasite population, odds ratio 2.85 (95% CI: 0.30–26.76)). There was no significant difference in fish mortalities between the parasite populations (Binary Logistic Regression: G = 5.125, d.f. = 3, p = 0.163): 16.1% mortality in pure and 25% in mixed populations.

## Discussion

This study unambiguously demonstrates the occurrence of sexual recombination in a monogenean parasite, *Gyrodactylus turnbulli*, by making experimental crosses and using a microsatellite marker to identify sexually derived parasites (i.e. outbred or hybrid genotype offspring). Although it has long been assumed that sex does occur in monogenean hermaphrodite parasites based on their observed inter- and intra-specific mating behaviour and phylogenetic studies [28], [31], [35], there was no direct evidence for the production of viable sexually produced offspring. Importantly, we also show that outcrossing (hybridisation) between monogeneans of previously inbred populations and/or inter-strain competition significantly increases various fitness components of the parasites, resulting in a higher parasite burden over time and an increased maximum parasite burden, but not a longer duration of infection.

Previously, cytogenetic observations of decondensation of the sperm nucleus after the fusion of sperm and egg cell had suggested that sexual recombination occurs in gyrodactylids [31]. However, release of male genetic material into the egg cytoplasm does not necessarily equate to sexual recombination since any sperm material can be expelled from the egg. Sperm dependent parthenogenesis, for example, is displayed by the hermaphrodite flatworm *Schmidtea polychroa* [42]. Further evidence of genetic exchange in the evolutionary history of gyrodactylids through sexual reproduction comes from previous phylogenetic studies (e.g. *G. salaris, Macrogyrodactylus* spp.) [14], [29]. However, the current study is the first to conclusively demonstrate sexual recombination in monogeneans in contemporary populations and establishes that, at least in the laboratory conditions of the present experiment, between 3.7% and 10.9% of all genotypes are recent hybrids with ancestors from two different inbred lines.

Confirmation of recombination in viviparous gyrodactylids has important consequences for the evolutionary history of this specious group of flatworms. Over 400 species of gyrodactylids have been described and host-switching rather than co-evolution with the fish host appears to be the main mechanism of speciation [43], [44]. Sexual reproduction between diverging populations can potentially facilitate the evolution of new species [7], [45], particularly in combination with a switch to a host not previously populated by either of the parental species [29], [46]. In more recent times, host switches and hybridisation events are further facilitated by changes in range distribution associated with climate change and global fish transport. The resulting co-occurrence of previously separated species in the same habitat [47], [48] and the new opportunities provided in these novel environments could augment the rate of hybridisation in the wild.

The occurrence and frequency of sexual reproduction in gyrodactylids may be species-specific and condition-dependent. Based on changes in haptor morphology over 20 generations of individually maintained parasites, Harris [49] speculated that *G. gasterostei* reproduction is largely clonal. As explained in the Material and Methods, our estimates of sexual reproduction for *G. turnbulli* are significantly downward biased given that we used only a single microsatellite locus to identify sexually reproduced offspring. Thereby, we will have missed hybrid genotype gyrodactylids that are homozygous for our marker locus due to, for instance, Mendelian segregation or back-crosses. The rates of sexual recombination varied by nearly a factor of three between our crosses. These differences may be due to the limitations of our methodology, or they could reflect natural variation in the rate of outcrossing.

Even a low frequency of sex in hermaphrodites is sufficient to avoid the negative fitness consequences associated with inbreeding [2]. Empirical data from the facultative sexual single-celled chlorophyte *Chlamydomonas reinhardtii* show that particularly in large hermaphrodite populations, sex will be maintained as a reproductive mode to overcome inbreeding depression [50]–[52]. Mate preference for unrelated individuals is likely to evolve as this reduces the fitness costs of sexual reproduction with a related individual. We previously speculated about inbreeding avoidance occurring in *Gyrodactylus* species [44] and empirical data from the current study is consistent with this argument, showing that the time-to-infection differs according to the relatedness of the novel gyrodactylid compared to the already existing infection. This suggests that these parasites may recognize conspecifics with similar genotypes. The mechanism for this remains unknown, but could be related to a chemical cue released through the parasite’s glands or excretory products into fish mucus [44]. Humans, mice and fish are known to use chemical communication (i.e. olfaction) to make self-referential decisions of attractiveness based on genetic variation at the Major Histocompatibility Complex (MHC) [53]. Many vertebrates show preferences for partners with dissimilar genotypes, which are identified via MHC-related chemical cues [54]. In invertebrates, behavioural mechanisms to avoid inbreeding are more commonly reported than the utilization of olfactory cues (e.g. insects: *Gryllus bimaculatus* and the subsocial spider, *Anelosimus* cf. *jucundus*) [55]–[57], but there is also evidence that the innate immune system may be involved in mate choice [54]. In the only other parasitic platyhelminth in which inbreeding avoidance has been examined, individuals of the cestode *Schistocephalus solidus* preferred siblings to unrelated parasites as mates [58]. Hence, to our knowledge, the current study provides the first evidence of inbreeding avoidance behaviour in platyhelminths.

Once sexual reproduction occurs, the main fitness benefit in hybridising gyrodactylid populations appears to be the higher maximum parasite burdens and extended time of population growth before the host’s immune response appears to cause an infra-population decline. Pure parasite populations started declining approximately seven days after initial infection, whereas mixed parasite infra-populations continued to grow on average for a further two days. These results are consistent with the hypothesis that: (i) hybrid genotypes are more tolerant (parasite damage limitation) and/or resistant (limitation of parasite burden) [59] to the fish immune response allowing them to maintain a reproducing population on the host for longer than parental parasites; (ii) hybrid genotypes are better at evading the host’s localised immune response (see van Oosterhout et al. for the effects of parasite mobility and distribution on parasite population dynamics) [60]; (iii) hybrid genotypes do not activate the immune response as rapidly as parental parasites, leading to delay in the onset of the host response; and/or (iv) they benefit from hybrid vigour (heterosis) and have increased fecundity relative to the inbred parental lines (see Cable & van Oosterhout for evidence of inbreeding depression in the *Gt3* line) [61]. Alternatively, the high fitness of mixed strain parasite populations could be caused by inter-strain competition, possibly resulting in increased virulence [62]. However, mixed parasite populations did not cause higher host mortalities in the current study, indicating that despite increased growth rates in parasite populations, virulence of hybrid genotypes was not increased compared to parental populations. Further evidence against inter-strain competition is the lack of evidence for competitive exclusion among other monogeneans co-infect the same host [63], [64].

The exact reasons for the increased fitness of mixed parasite populations cannot be disentangled in this experiment, but warrant further study. Given the reduction in fitness of the *Gt3* line relative to recently wild-caught *G. turnbulli* (*Gt1*) [61], we favour the hypothesis that hybridisation ameliorated the detrimental effects of inbreeding during the up to ∼2×10^3^ generations in captivity, and that this resulted in hybrid vigour. There are implications of this study for the fitness of hosts and parasites in wild populations. In natural environments, a high parasite burden is associated with increased extrinsic mortality of this host, for example due to increased risk of downstream displacement during flash-flooding [65]. This suggests that the increased parasite load in hybrid genotype infections could increase host mortality in the wild. Similarly, secondary (bacterial, fungal, etc.) infections may be more common with higher parasite burdens. In addition, other factors such as infectivity, transmission abilities and host specificity, which have not been considered for this experiment, could also be affected by hybrid vigour, and the effects of hybridisation on these life history traits and infection dynamics warrant further investigations.
